# Case report: Trauma-induced *Klebsiella pneumoniae* invasive syndrome presenting with liver abscess, lung abscess, endophthalmitis, and purulent meningitis

**DOI:** 10.3389/fmed.2024.1513831

**Published:** 2025-01-07

**Authors:** Hong-qiao Chen, Zhen-hua Mo, Wu-xiao Wei

**Affiliations:** Department of Neurology, Guangxi University of Science and Technology First Affiliated Hospital, Liuzhou, China

**Keywords:** *Klebsiella pneumoniae* invasive syndrome, trauma-induced infection, liver abscess, lung abscess, endophthalmitis, purulent meningitis, diabetes mellitus, case report

## Abstract

**Purpose:**

This study aims to explore the underlying causes, diagnostic strategies, and treatment approaches of trauma-induced *Klebsiella pneumoniae* invasive syndrome (KPIS) through a rare case report. By highlighting the role of trauma as a potential trigger for KPIS, particularly in high-risk populations such as individuals with diabetes, this study seeks to provide valuable insights for improving clinical outcomes and promoting public health awareness.

**Background:**

*Klebsiella pneumoniae* invasive syndrome is a multi-organ infectious disease commonly associated with complications such as liver abscess, lung abscess, endophthalmitis, and purulent meningitis, with high mortality and disability rates. In recent years, the incidence of KPIS has been increasing, particularly in the Asia-Pacific region, and is closely linked to hypervirulent *Klebsiella pneumoniae* (hvKp) infections. While extensive research has focused on the risk of KPIS in patients with underlying conditions such as diabetes, trauma-induced KPIS remains exceedingly rare, with limited understanding of its pathophysiology and clinical management. Trauma may facilitate invasive infections by disrupting immune barriers and compromising local tissue integrity, creating entry points for pathogens.

**Case presentation:**

This study reports a case of a 72-year-old male who developed multiple infections, including liver abscess, lung abscess, left endophthalmitis, and purulent meningitis, following a traumatic fall. The patient had a history of poorly controlled diabetes mellitus. A diagnosis of KPIS caused by hvKp was confirmed through bacterial cultures and a positive string test. Imaging studies revealed multi-organ involvement. Given the complexity of the patient’s condition, a comprehensive treatment regimen, including broad-spectrum antibiotics, was implemented with significant therapeutic success. The patient showed marked improvement and continued follow-up after discharge, with a notable resolution of the infections.

**Conclusion:**

This case highlights the significance of trauma as a potential trigger for KPIS, particularly in patients with high-risk underlying conditions such as diabetes. Early recognition and the implementation of individualized anti-infective treatment are crucial for reducing mortality and improving prognosis in KPIS patients. Future research should further investigate the relationship between trauma and hvKp infections and develop more comprehensive diagnostic and therapeutic guidelines.

## Introduction

*Klebsiella pneumoniae* invasive syndrome (KPIS) is a rare but increasingly recognized infectious disease, characterized by invasive infection of multiple organs, including the liver, lungs, eyes and central nervous system. The majority of KPIS cases are related to highly pathogenic strains of *Klebsiella pneumoniae* (Kp), which can spread through the bloodstream and cause serious complications, such as liver abscess, lung abscess, endophthalmitis and purulent meningitis (1). It is more common in individuals with diabetes, hepatobiliary diseases, pancreatic diseases, or those with immunosuppression (2–4). Recently, the incidence of KPIS has increased, especially in the Asia-Pacific region, and highly pathogenic strains account for a large part of cases (5). Despite the increasing recognition of KPIS, cases caused by trauma are still very rare. Trauma can weaken immune defenses, provide entry points for pathogens, and trigger invasive infections in susceptible individuals (6). However, the relationship between trauma and KPIS is not well-documented, which makes the diagnosis and management of this situation particularly challenging.

## Case presentation

**FIGURE 1:**
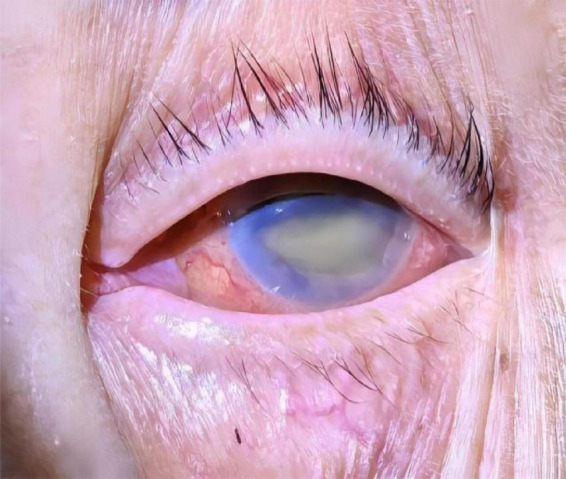
Results of ocular examinations. The left conjunctiva appeared hyperemic, the left cornea was white and cloudy, and massive pus accumulation was observed in the anterior chamber, suggesting left endophthalmitis as a secondary infection.

A 72-year-old male patient from Liuzhou, Guangxi Zhuang Autonomous Region, was hospitalized due to 12 days of right frontal pain following a fall, accompanied by dyspnea and abnormal mental behavior that developed 11 h prior to admission. According to his family, the patient fell while exercising 12 days earlier, experiencing right frontal pain, headache, and dizziness. However, he did not seek medical attention, opting to manage the injury independently. Eleven hours before hospitalization, he developed dyspnea and abnormal mental behavior, prompting a visit to the emergency department of our hospital. There, he received sedation and non-invasive ventilator-assisted respiration. The patient had a history of type 2 diabetes with recent poor glycemic control but no history of travel, respiratory disease, or urinary tract infection in the 6 months prior to admission. There is no similar medical history in the family. On admission, his physical examination revealed the following: T 38.3°C, P 138 bpm, BP 121/75 mmHg (1 mmHg = 0.133 kPa), SpO2 95%, and a sedated state under non-invasive ventilator-assisted respiration. A contusion and laceration approximately 4 cm × 0.5 cm were observed on the right forehead, with local redness, swelling, and slight suppurative secretion. The skin surface was warm with a sensation of fluctuation. His pupils were unequal in size; the left pupil measured 3 mm and was non-reactive to light, while the right pupil measured 2 mm and was poorly reactive to light. The left eyelid and conjunctiva were congested, with massive pus accumulation in the anterior chamber. Further examination showed symmetrical bilateral nasolabial grooves with no distortion of the commissure, shortness of breath, and respiratory harshness in both lungs, along with slight moist rales bilaterally. Neurological examination revealed a stiff neck, three transverse fingers, and negative Kernig and Brudzinski signs. Ocular examination revealed hyperemia of the left eyelid and conjunctiva, a white and cloudy left cornea, massive pus accumulation in the anterior chamber, and an invisible iris, suggesting left endophthalmitis as a secondary infection (Figure 1).

**FIGURE 2:**
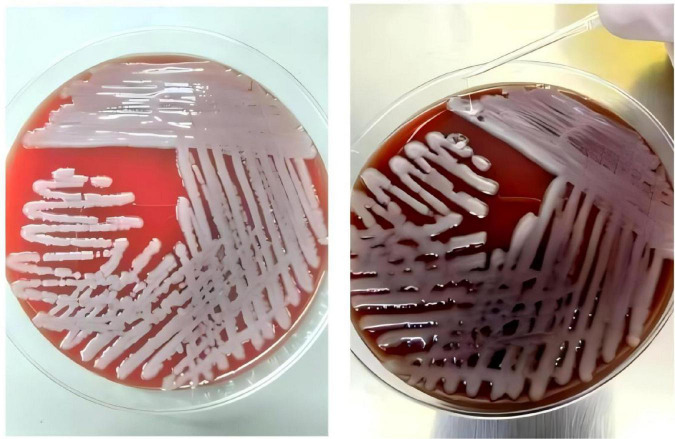
Blood agar plate: positive string test for the *Klebsiella pneumoniae* isolated from the present patient.

Complete blood count showed WBC 13.58 × 10^9^/L (normal range: 3.5–9.5 × 10^9^/L), NE% 94.1% (normal range: 40–75%), HGB 119 g/L (normal range: 130–175 g/L), and PLT 374 × 10^9^/L (normal range: 125–350 × 10^9^/L). CRP was 8.9 mg/L (normal range: 0–10 mg/L). Liver function tests revealed TBIL 103.3 μmol/L (normal range: 3–21 μmol/L), DBIL 62.83 μmol/L (normal range: 0–6.8 μmol/L), IBIL 40.5 μmol/L (normal range: 0–17 μmol/L), AST 61.3 U/L (normal range: 0–40 U/L), and ALT 79.5 U/L (normal range: 0–50 U/L). Blood gas analysis indicated pH 7.39 (normal range: 7.35–7.45), PaO_2_ 95 mmHg (normal range: 80–100 mmHg), PCO_2_ 21 mmHg (normal range: 35–45 mmHg), ${\text{HCO}}_{\text{3}}^{\text{−}}$ 12.7 mmol/L (normal range: 21–27 mmol/L), and lactic acid 1.9 mmol/L (normal range: 0.9–1.7 mmol/L). Procalcitonin (PCT) was elevated at 43.13 ng/mL (normal range: 0–0.05 ng/mL), and IL-6 was 1515.00 pg/mL (normal range: 0–6.6 pg/mL). Erythrocyte sedimentation rate (ESR): 38 mm/h (normal range: 0–15 mm/h). Blood glucose was 15.4 mmol/L (normal range: 3.9–6.1 mmol/L). Kp was detected in pus secretion, blood culture, and cerebrospinal fluid (CSF) culture, while no fungi were detected. A positive string test confirmed the hypervirulent strain of Kp (Figure 2). Lumbar puncture revealed yellow, cloudy, and purulent CSF at a pressure of 150 mmH_2_O (normal range: 70–200 mmH_2_O). CSF analysis showed WBC 448 × 10^6^/L (normal range: 0–10 × 10^6^/L), multinuclear cell percentage 96.2%, mononuclear cell percentage 3.8%, Cl^–^ 134.13 mmol/L (normal range: 120–130 mmol/L), glucose 2.16 mmol/L (normal range: 2.5–4.5 mmol/L), and protein level 1761.51 mg/L (normal range: 0–430 mg/L). No significant abnormalities were found in other tests.

**FIGURE 3:**
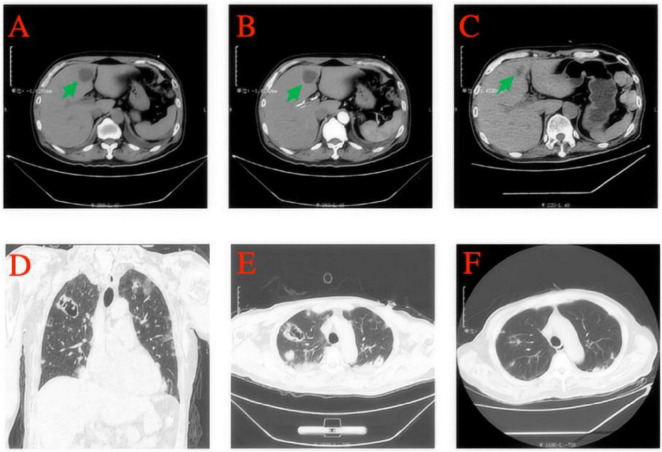
A whole-abdomen CT scan, including plain and enhanced views, revealed a low-density lesion approximately 28 mm in diameter in the S4 segment of the left liver lobe, with a well-defined boundary and a CT value of 16 HU **(A)**. Enhanced imaging further demonstrated non-homogeneous enhancement and large patchy halo signs, consistent with a diagnosis of a liver abscess **(B)**. After more than 6 weeks of combined antibiotic therapy, significant shrinkage of the liver abscess was observed **(C)**. Chest CT scans revealed scattered nodular and patchy blurred shadows with increased density in both lungs, with some lesions showing cavitation, indicative of bilateral pneumonia and multiple lung abscesses **(D,E)**. A subsequent follow-up CT demonstrated marked improvement, with substantial absorption of pulmonary lesions in both lungs **(F)**.

Imaging studies included a whole abdomen CT scan with plain and enhanced views, which showed a low-density lesion approximately 28 mm in diameter in the S4 segment of the left liver lobe, with a clear boundary and a CT value of 16 HU. Enhanced imaging revealed significant non-homogeneous enhancement with large patchy halo signs, indicative of a liver abscess (Figures 3A, B). Chest CT scans revealed scattered nodular and patchy blurred shadows with increased density in both lungs, some of which were cavitary, suggestive of bilateral pneumonia with multiple lung abscesses (Figures 3D, E). After more than 6 weeks of combined antibiotic therapy, follow-up chest and abdominal CT scans showed significant absorption and reduction of bilateral pulmonary lesions. The liver lesion in the S4 segment of the left liver lobe also decreased significantly in size (Figures 3C, F).

The diagnosis of trauma-induced KPIS was challenging due to its rare etiology, non-specific symptoms, and multi-organ involvement, including liver abscess, lung abscess, endophthalmitis, and purulent meningitis. The lack of advanced diagnostic tools further complicated the identification of the hypervirulent strain. However, a combination of clinical history (trauma and poorly controlled diabetes), positive Kp cultures from multiple sites, imaging findings, and the positive string test ultimately confirmed the diagnosis. Based on the susceptibility report for Kp (Table 1), he received an anti-infection regimen of meropenem (2000 mg IV q8h) combined with cefoperazone sulbactam sodium (1 g IV q8h). The patient was hospitalized for a total of 54 days, and after discharge, began oral treatment with ceftriaxone (2 g/day). The patient is currently under regular follow-up.

**TABLE 1:** Patient’s susceptibility report.

Antibiotic	Susceptibility	MIC (μ g/mL)
Piperacillin-tazobactam	S	≤4
Cefuroxime	S	2
Cefoxitin	S	≤4
Ceftazidime	S	≤0.12
Ceftriaxone	S	≤0.25
Cefoperazone sulbactam	S	≤8
Cefepime	S	≤0.12
Amikacin	S	≤2
Imipenem	S	≤0.25
Ertapenem	S	≤0.12
Meropenem	S	≤0.25
Levofloxacin	S	≤0.12

S, susceptible; MIC, minimum inhibitory concentration.

## Discussion

*Klebsiella pneumoniae* invasive syndrome was first discovered in 1986 in Taiwan and was subsequently reported in Europe, the United States, and Australia, although the Asia-Pacific region has remained the major area of incidence (1). Among these, K1 and K2 are recognized as the most virulent and common capsular serotypes, often exhibiting high viscosity, as demonstrated by a positive string test. Over recent decades, the infection rate of hypervirulent *Klebsiella pneumoniae* has gradually increased. HvKp is characterized by its ability to infect healthy individuals and cause high mortality rates, posing a significant challenge to the prevention, control, and management of infected patients (1, 7, 8).

Trauma is a significant predisposing factor for bloodstream infections caused by Kp. Disruption of skin and mucosal barriers during trauma facilitates the entry of pathogens into the bloodstream, leading to invasive infections. Moreover, trauma often necessitates invasive medical interventions, such as catheterization and mechanical ventilation, which increase the risk of nosocomial infections (9). At the molecular level, trauma triggers the release of pro-inflammatory cytokines, such as IL-6 and TNF-α, which paradoxically impair the phagocytic activity of immune cells. This immune dysregulation, coupled with local tissue damage, promotes bacterial colonization and dissemination (10).

In diabetic patients, hvKp is the main pathogen of liver abscess. This is probably because hyperglycemia promotes the hvKp colonization in the intestinal system, while the relatively low immunity of diabetic patients makes the bacterium easily pass through the portal system and break through the blood-brain and blood-eye barriers, consequently resulting in multiple systemic infections such as liver abscess, lung abscess, endophthalmitis and suppurative meningitis (8). The clinical symptoms of Kp-related invasive liver abscess syndrome are non-specific, often presenting with fever, chills, and abdominal pain. The infection occasionally extends to extrahepatic areas like the eyes, lungs, and central nervous system, causing related symptoms (11). Due to its latent nature, the disease is often misdiagnosed or missed in clinical practice, preventing early detection and treatment, which negatively impacts the prognosis.

HvKp liver abscess differs from other etiologies, such as Aspergillus and amebic liver abscesses, in several key ways. It is frequently linked to diabetes and can result in metastatic complications like endophthalmitis and meningitis due to the bacterium’s capacity to breach organ barriers. In contrast, Aspergillus liver abscess is primarily seen in immunocompromised individuals, particularly those undergoing chemotherapy or organ transplantation, and is often associated with disseminated fungal infections (12). On the other hand, amebic liver abscess, caused by Entameba histolytica, typically occurs in individuals residing in or traveling to endemic regions and is characterized by fever and right upper quadrant pain without metastatic complications (13). The diagnostic approaches for these etiologies differ significantly. hvKp liver abscess diagnosis relies on bacterial culture and imaging, often demonstrating hypervirulent strains through a positive string test. Aspergillus liver abscess requires fungal cultures, serum galactomannan (GM) testing, or histopathological analysis, while amebic liver abscess is confirmed through serology or PCR.

The patient had a history of trauma, which suggested trauma-induced KPIS. Bacterial serotyping, bacterial-associated virulence factor testing, and next-generation sequencing (NGS) facilitate the early and accurate diagnosis of KPIS and are also useful for post-diagnosis analysis of drug resistance (14, 15). Unfortunately, due to personal and family financial constraints, these tests were not performed for this patient.

Currently, there are no clear guidelines for treating KPIS. In addition to strict blood glucose control, early administration of an adequate dose and a sufficient course of intravenous antibiotics, such as quinolones, 3rd or 4th generation cephalosporins, aminoglycosides, and carbapenems, is often required. The course of antibiotic therapy usually lasts 4–6 weeks, with the duration depending on the lesion control (1). Major treatments for KIPS include medication, local drainage and surgery. For liver abscesses <3 cm in diameter or early partially liquefied abscesses, medication is generally adopted. For fully liquefied liver abscesses >3 cm, ultrasound-guided percutaneous aspiration is applied (16). The liver lesion of the patient in this study was an abscess about 2.8 cm in size. Drainage of a small liver abscess may lead to complications like capsular blockage-induced peritonitis diffusion, resulting in adverse outcomes. We did not choose percutaneous pus aspiration. After combined antibiotic therapy, the liver abscess was significantly reduced, achieving effective treatment. In the case of lung abscesses, conservative medical management is usually adopted, including antibiotics and chest physiotherapy. For patients with a lung abscess cavity larger than 6 cm where conservative management fails, invasive surgery (usually pulmonary lobectomy) or percutaneous pus drainage may be considered (17). Endophthalmitis is a serious vision-threatening complication of KPIS, leading to permanent vision loss in 25.8% (5–50%) of cases. Its treatments primarily include intravitreal injection and vitrectomy (18). Apart from systemic antibiotic therapy, intravitreal steroids are also effective in saving a patient’s vision, and where medication is ineffective, vitrectomy may be considered (19). Suppurative meningitis is rare in KPIS, but when it occurs, it is often accompanied by cerebrovascular complications, such as subarachnoid hemorrhage, intracranial hemorrhage, and venous sinus thrombosis. In addition to antibiotics, adjuvant therapy with dexamethasone and mannitol can also be adopted according to the patient’s condition (20). In addition, treating patients with severe multidrug-resistant (MDR) infections in an intensive care setting presents many challenges.

## Conclusion

This case highlights the need for increased health awareness regarding traumatic infections, especially among patients with chronic conditions like diabetes. Early detection and prompt management of KPIS and its complications are essential for improving long-term outcomes and reducing mortality rates.

## Data Availability

The original contributions presented in this study are included in this article/supplementary material, further inquiries can be directed to the corresponding author.
